# Fecal Impaction and Nonperforated Stercoral Colitis: Red Flags for Poor Outcomes

**DOI:** 10.7759/cureus.41705

**Published:** 2023-07-11

**Authors:** Michael Sacerdote, Joseph Limback, Jianbin Zhu

**Affiliations:** 1 Radiology, AdventHealth Orlando, Orlando, USA; 2 Biostatistics, AdventHealth Orlando, Orlando, USA

**Keywords:** mortality risk, stercoral perforation, stercoral ulcer, geriatrics, hospital outcomes, stercoral colitis, fecal impaction

## Abstract

Fecal impaction and stercoral colitis are common, yet little research has been performed on the associated mortality risk. We performed a retrospective cohort study of 970 hospital encounters representing 885 unique patients in which fecal impaction or stercoral colitis was identified in CT reports. Among the 535 patients with fecal impaction, 13.3% died or were discharged to hospice, compared to 13.1% among the 428 patients with nonperforated stercoral colitis (p = 0.93). Of the seven patients with perforation, five died or were discharged to hospice. The risk of death or discharge to hospice for patients with fecal impaction or nonperforated stercoral colitis aged 18-49 was 2.9% and rose approximately 4% each decade thereafter to 21.9% for patients 90 and older (p< 0.001). Patients with a body mass index of 25-30 had an 8.1% risk of death or discharge to hospice, compared to 23.4% for those with a BMI < 18.5 (p< 0.001). Patients with at least one ICD-10 code for dementia, paralysis/neuromuscular disease, or malnutrition/failure to thrive had a risk of death or discharge to hospice of 21.6%, compared to 1.9% among patients with none of these risk factors (p< 0.001). ICD-10 codes for sepsis were associated with 90.0% of the deaths and 44.3% of the discharges to hospice. Patients diagnosed in less than three hours had a risk of death or discharge to hospice of 8.0%, compared to a risk of 20.1% for those diagnosed in ≥ 12 hours (p< 0.001).

## Introduction

Fecal impaction is a “large hard inspissated concreted stool or bezoar lodged in the lower GI tract” that the patient is unable to evacuate [1]. Stercoral colitis is colonic inflammation secondary to fecal impaction [2]. Stercoral ulcers are erosions of the colon caused by impacted feces [3]. Stercoral perforations are bowel perforations due to pressure necrosis from fecal masses [4].

Fecal impaction and stercoral colitis are both extremely common. Read reported in 1985 (without presenting the underlying data) that 42% of all patients admitted to a geriatric ward in the UK had fecal impaction [5]. Fecal impaction diagnosed by rectal exam had a prevalence of 6.6% among 665 Spanish nursing home residents and a one-year incidence of 47.3% [6]. The ICD-9 code for fecal impaction was present in 42,481 ED visits in the US in 2011 [7]. Stercoral perforation of the colon accounted for 1.2% of all emergency colorectal procedures and 3.2% of colonic perforations seen at a Swiss hospital from 1993 to 1998 [8].

Despite the frequency with which fecal impaction and stercoral colitis are encountered in practice, the literature on these diseases is sparse, and the vast majority of papers on the subject consist of case reports and case series with 10 or fewer patients. A review of 188 case reports and short case series describing fecal impaction and stercoral colitis in 280 patients up to June 2014 found that 28% of cases reported resulted in death [9]. The most common complications were perforation (52%), intestinal obstruction (13%), stercoral ulcer (11%), and obstructive uropathy (10%). A review of 24 case reports and case series describing 56 patients with stercoral perforation occurring in 1998-2011 found that patients died in 34% of cases [10]. While case reports typically describe exceptional or severe cases, the implication is that serious complications associated with fecal impaction and stercoral colitis are relatively common.

Aside from small case series, there are only a handful of systematically performed studies on outcomes associated with fecal impaction and stercoral colitis. The first large study of fecal impaction consisted of a retrospective study of 146 cases (130 unique patients) in Beirut discharged from the hospital or emergency room with a diagnosis of fecal impaction from 1992 to 2009 [11]. The diagnosis was made by digital rectal exam in 36%, imaging in 24%, digital rectal exam and imaging in 39%, and intraoperatively in 1%; the type of imaging used for diagnosis was not disclosed. The mean age was 67.1 years, with 61% women. Infectious complications occurred in 11%. One patient died.

A group at Harvard Medical School retrospectively identified 42 ED visits (32 unique patients) with the ICD-10 code for fecal impaction (K56.41) in 2016 and 2017 [12]. The mean age was 72.9 and 63% were women. The patients averaged 8.7 medical diagnoses and 11 medications; almost half came from institutionalized settings. Seven patients died, with four deaths “plausibly related to/secondary to fecal impaction.”

A Turkish group retrospectively identified 41 patients with stercoral colitis admitted from 2006 to 2015 [13]. The group was vague about how the patients were identified, but all patients had fecalomas with colon distention of >6 cm, colonic wall thickening of >3 mm, and pericolonic fat stranding. The median age was 74, 54% were women, and “there was no significant relationship between the age of the patients and mortality.” Chronic diseases were common. Ten of the patients (24%) died.

A Korean group published an abstract describing 24 patients with stercoral colitis diagnosed by imaging or endoscopically who presented with unstable vital signs or multiorgan failure from 2010 to 2018 [14]. The median age was 81.8, 75% were women, and mortality was 46% despite only five (20.8%) having perforation.

A group at the Mayo Clinic retrospectively identified 269 ED patients (median age 76, 53% female) with stercoral colitis based on a keyword search of radiology reports from 2018 to 2021 [15]. All-cause mortality among all ED patients with stercoral colitis was 7.8% within one month and 10.8% within one month among those admitted to the hospital.

We aimed to measure the mortality risk among patients with radiologically suspected fecal impaction and stercoral colitis; compare outcomes between fecal impaction and nonperforated stercoral colitis; identify risk factors associated with poor outcomes; and gather additional data that could be used for future hypothesis generation.

## Materials and methods

The AdventHealth Institutional Review Board (IRB) (1668944-9) approved this study. Keyword searches of radiology reports were performed by our departmental IT staff. The data were collected and analyzed by two board-certified radiologists. A professional biostatistician performed the statistical tests.

Patients

We performed a retrospective cohort study of patients with radiologically suspected fecal impaction and/or stercoral colitis drawn from a hospital system in the southeast, including a 1,400-bed tertiary hospital and 12 small community hospitals.

**Figure 1:**
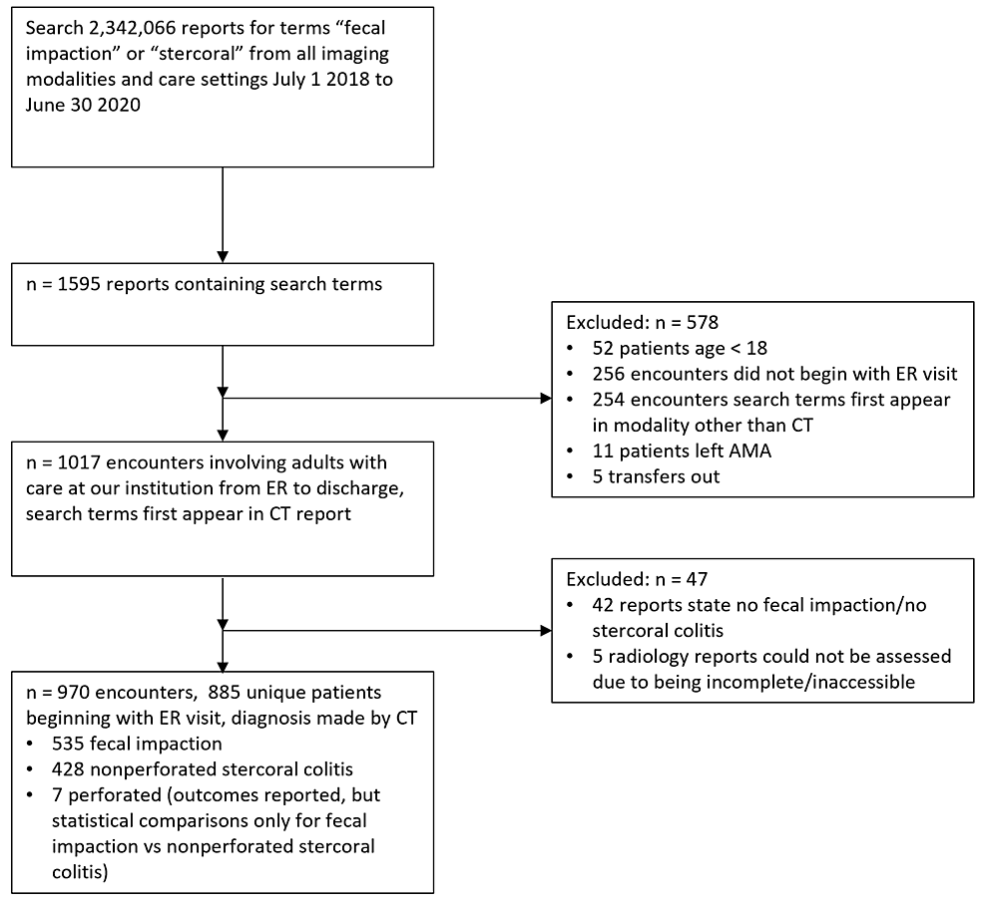
Inclusion/exclusion flowsheet

We searched 2,342,066 reports from all imaging modalities dated July 1, 2018 to June 30, 2020 for the search terms “fecal impaction” and “stercoral.” 1595 imaging reports contained at least one of the search terms. We excluded 52 patients who were under 18 years of age, 256 patients whose encounter did not begin with an ED visit (such as outpatient visits, transfers from outside hospitals, and direct admissions that bypassed the ED), 254 patients where the search terms first appeared in a study from a modality other than CT, 11 patients who left against medical advice, five patients who were transferred out to other hospitals, and five patients where the radiology reports were incomplete or inaccessible. Of the remaining 1,012 patients, we reviewed the CT reports and excluded 42 patients in which the report stated that fecal impaction or stercoral colitis was absent. These filters yielded 970 encounters representing 885 unique adult patients whose hospital encounter began in the ED and who were diagnosed with at least possible fecal impaction or stercoral colitis by CT. Our inclusion/exclusion criteria flowsheet is presented in Figure 1.

We recorded the race documented in the chart; at the time of the study, ethnicity was not stored in our medical records system. Height and weight were the values either measured or estimated by the triage nurse. If the height and weight were not measured or estimated at the time of triage, they were listed as “not recorded.”

Clinical course

Our primary outcome was combined in-hospital mortality or discharge to hospice. We measured the number of discharges from the ED, hospital admissions, ICU admissions, length of stay, in-hospital mortality, and discharges to hospice. Discharge to hospice included in-hospital hospice, hospice at dedicated facilities, and home hospice. “Do not resuscitate” status was not counted as hospice. In-hospital mortality was counted if the patient expired in the hospital during the same encounter as the CT report containing the search terms.

We collected all ICD-10 codes associated with each encounter. For each of the 72,836 valid ICD-10 codes, we counted the number of occurrences in our dataset and how often each ICD-10 code appeared in each group of patients. Patients were identified as having dementia, sepsis, diabetes, decubitus ulcers, paralysis/neuromuscular disease, or malnutrition/failure to thrive based on ICD-10 codes. See Table 1 for the lists of ICD-10 codes used for each diagnostic group.

We assessed whether patients had been evaluated by consultants specializing in gastroenterology, general or colorectal surgery, and infectious diseases. A consultation was counted if the consultant left a note in the chart or if a consultation was recorded in the discharge summary; telephone conversations between ED providers and specialists where the consultant did not personally evaluate the patient were not counted. We assessed whether patients underwent esophagogastroduodenoscopy, colonoscopy, or any abdominal surgical procedure. We included all gastrointestinal tract surgery, percutaneous endoscopic gastrostomy tubes, gastrostomy tubes placed by interventional radiology, hepatobiliary surgery, gynecologic surgery, and exam under anesthesia in surgical procedures, but we did not include vascular procedures, orthopedic procedures, or debridement of decubitus ulcers.

**Table 1:** ICD-10 code groups ICD-10 codes with at least one occurrence in our dataset were aggregated into the groups as listed in this table. We then searched the list of ICD-10 codes associated with each patient encounter for the presence of codes from each group. Codes are ordered in this table by descending frequency.

ICD-10 codes	Short Description
Dementia and Dementing Diseases	
F03.90	Unsp dementia, unsp severity, without beh/psych/mood/anx
G30.9	Alzheimer's disease, unspecified
G20	Parkinson’s disease
F01.50	Vascular dementia, unsp severity, without beh/psych/mood/anx
F01.51	Vascular dementia with behavioral disturbance
G31.83	Neurocognitive disorder with Lewy bodies
F02.81	Dementia in oth diseases classd elswhr w behavioral disturb
G23.1	Progressive supranuclear ophthalmoplegia
G30.1	Alzheimer's disease with late onset
G31.01	Pick's disease
Paralysis/Neuromuscular Disease	
R53.2	Functional quadriplegia
G20	Parkinson's disease
I69.35	Hemiplga following cerebral infrc affecting left nondom side
I69.351	Hemiplga following cerebral infrc aff right dominant side
G80.9	Cerebral palsy, unspecified
G35	Multiple sclerosis
G82.2	Paraplegia, unspecified
Q05.9	Spina bifida, unspecified
G85.50	Quadriplegia, unspecified
G81.91	Hemiplegia, unspecified affecting right dominant side
G12.21	Amyotrophic lateral sclerosis
I69.39	Hemiplga following cerebral infarction affecting unsp side
G80.0	Spastic quadriplegic cerebral palsy
G72.81	Critical illness myopathy
I69.954	Hemiplga fol unsp cerebvasc disease aff left nondom side
G80.8	Other cerebral palsy
G81.91	Hemiplegia, unspecified affecting right dominant side
I69.365	Oth paralytic syndrome following cerebral infrc, bilateral
G70.01	Myasthenia gravis with (acute) exacerbation
G72.41	Inclusion body myositis [IBM]
G72.9	Myopathy, unspecified
G81.90	Hemiplegia, unspecified affecting unspecified side
G81.92	Hemiplegia, unspecified affecting left dominant side
G83.4	Cauda equina syndrome
G83.9	Paralytic syndrome, unspecified
G95.20	Unspecified cord compression
G95.29	Other cord compression
I69.265	Oth paralytic syndrome following oth ntrm intcrn hemor, bi
I69.341	Monoplg low lmb fol cerebral infrc aff right dominant side
I69.851	Hemiplga fol oth cerebvasc disease aff right dominant side
I69.951	Hemiplga fol unsp cerebvasc disease aff right dominant side
I69.965	Oth paralytic syndrome following unsp cerebvasc disease, bi
Malnutrition	
E43	Unspecified severe protein-calorie malnutrition
R62.7	Adult failure to thrive
E46	Unspecified protein-calorie malnutrition
E44.0	Moderate protein-calorie malnutrition
R64	Cachexia
R63.0	Anorexia
R63.4	Abnormal weight loss
R63.6	Underweight
E44.1	Mild protein-calorie malnutrition
Diabetes	
E11.9	Type 2 diabetes mellitus without complications
E11.22	Type 2 diabetes mellitus w diabetic chronic kidney disease
E11.65	Type 2 diabetes mellitus with hyperglycemia
E11.51	Type 2 diabetes w diabetic peripheral angiopath w/o gangrene
E11.42	Type 2 diabetes mellitus with diabetic polyneuropathy
E11.69	Type 2 diabetes mellitus with other specified complication
E11.649	Type 2 diabetes mellitus with hypoglycemia without coma
E11.40	Type 2 diabetes mellitus with diabetic neuropathy, unsp
E11.10	Type 2 diabetes mellitus with ketoacidosis without coma
E11.621	Type 2 diabetes mellitus with foot ulcer
E11.21	Type 2 diabetes mellitus with diabetic nephropathy
E11.52	Type 2 diabetes w diabetic peripheral angiopathy w gangrene
E11.43	Type 2 diabetes w diabetic autonomic (poly)neuropathy
E10.9	Type 1 diabetes mellitus without complications
E11.319	Type 2 diabetes w unsp diabetic rtnop w/o macular edema
E11.628	Type 1 diabetes mellitus with other skin complications
E13.10	Oth diabetes mellitus with ketoacidosis without coma
E11.00	Type 2 diab w hyprosm w/o nonket hyprgly-hypros coma (NKHHC)
E11.8	Type 2 diabetes mellitus with unspecified complications
E13.9	Other specified diabetes mellitus without complications
E09.22	Drug/chem diabetes w diabetic chronic kidney disease
E09.51	Drug/chem diabetes w diabetic prph angiopath w/o gangrene
E10.649	Type 1 diabetes mellitus with hypoglycemia without coma
E10.65	Type 1 diabetes mellitus with hyperglycemia
E11.11	Type 2 diabetes mellitus with ketoacidosis with coma
E11.39	Type 2 diabetes w oth diabetic ophthalmic complication
E11.41	Type 2 diabetes mellitus with diabetic mononeuropathy
E11.49	Type 2 diabetes w oth diabetic neurological complication
E11.59	Type 2 diabetes mellitus with oth circulatory complications
E11.622	Type 2 diabetes mellitus with other skin ulcer
Decubitus Ulcers	
L89.159	Pressure ulcer of sacral region, unspecified stage
L89.154	Pressure ulcer of sacral region, stage 4
L89.153	Pressure ulcer of sacral region, stage 3
L89.152	Pressure ulcer of sacral region, stage 2
L89.90	Pressure ulcer of unspecified site, unspecified stage
L89.150	Pressure ulcer of sacral region, unstageable
L89.899	Pressure ulcer of other site, unspecified stage
L89.610	Pressure ulcer of right heel, unstageable
L89.619	Pressure ulcer of right heel, unspecified stage
L89.214	Pressure ulcer of right hip, stage 4
L89.323	Pressure ulcer of left buttock, stage 3
Sepsis	
A41.9	Sepsis, unspecified organism
R65.21	Severe sepsis with septic shock
R65.20	Severe sepsis without septic shock
A41.51	Sepsis due to Escherichia coli [E. coli]
A41.59	Other Gram-negative sepsis
A41.02	Sepsis due to Methicillin resistant Staphylococcus aureus
A41.89	Other specified sepsis
A40.1	Sepsis due to streptococcus, group B
A40.8	Other streptococcal sepsis
A41.01	Sepsis due to Methicillin susceptible Staphylococcus aureus
A41.50	Gram-negative sepsis, unspecified
A41.81	Sepsis due to Enterococcus
A41.2	Sepsis due to unspecified staphylococcus
A41.4	Sepsis due to anaerobes
B37.7	Candidal sepsis
T81.44XA	Sepsis following a procedure, initial encounter

Statistics

Two board-certified radiologists reviewed the charts and encoded the data in a standardized spreadsheet. All data points were present in every chart except for race, height, and weight. 10% of the encounters were selected at random and the data abstractors provided independent duplicate data on these 97 encounters for the data points that were not copy-pasted electronically from the medical charts [16]. The inter-rater reliability kappa statistic was 0.89 or higher for each variable.

Chi-squared tests were used to test the associations of fecal impaction and nonperforated stercoral colitis with categorical outcomes (admitted, ICU stay, died, discharged to hospice, and combined died or discharged to hospice) and ICD-10 codes. The Mann-Whitney U test was used for the continuous outcome of length of stay. Univariate logistic regression models were used to find the odds ratios of demographics and ICD-10 code groups with the outcome of death or discharged to hospice. We did not include the seven patients with perforation in the statistical tests due to the small number of cases.

## Results

During the study period, there were 463,946 radiology studies of the abdomen and pelvis of all modalities, of which 201,076 were CT scans of the abdomen and pelvis. Of the 970 encounters meeting the study criteria, 535 CT reports included fecal impaction without mention of stercoral colitis or perforation, 428 CT reports included stercoral colitis with or without mention of fecal impaction but without mention of perforation, and seven CT reports included perforation with mention of fecal impaction, stercoral colitis, or both. For the rest of the paper, we will refer to these groups as "fecal impaction," "nonperforated stercoral colitis," and "perforated."

Patients meeting the inclusion criteria accounted for 0.48% of CT scans of the abdomen and pelvis performed during the study period. Patients with fecal impaction accounted for 0.27% of the CT scans of the abdomen and pelvis, patients with nonperforated stercoral colitis accounted for 0.21%, and perforated impaction/stercoral colitis accounted for 0.003%. Demographics are listed in Table 2.

**Table 2:** Demographics Patient demographics by gender, age, race, and BMI are shown above and are given on an encounter basis, not as the number of unique patients.  Table entries are formatted as N (%), except for the row labeled "Number of Encounters" and rows with average and median values.  Percentages apply to columns using the entries in the row labeled "Number of Encounters" as the denominators.

	Total, N (%)	Fecal Impaction, N (%)	Nonperforated Stercoral Colitis, N (%)	Perforated, N (%)
Number of Encounters	970	535	428	7
Gender				
Male	413 (42.6%)	238 (44.5%)	174 (40.7%)	1 (14.3%)
Female	557 (57.4%)	297 (55.5%)	254 (59.3%)	6 (85.7%)
Age				
Average (median)	70.6 (75)	71.6 (76)	69.4 (74)	72.4 (69)
18-49	139 (14.3%)	78 (14.6%)	61 (14.3%)	0 (0%)
50-59	102 (10.5%)	52 (9.7%)	50 (11.7%)	0 (0%)
60-69	123 (12.7%)	61 (11.4%)	58 (13.6%)	4 (57.1%)
70-79	243 (25.1%)	122 (22.8%)	120 (28.0%)	1 (14.3%)
80-89	249 (25.7%)	146 (27.3%)	101 (23.6%)	2 (28.6%)
≥90	114 (11.8%)	76 (14.2%)	38 (8.9%)	0 (0%)
Race				
White	666 (68.7%)	373 (69.7%)	288 (67.3%)	5 (71.4%)
Black	144 (14.5%)	77 (14.4%)	66 (15.4%)	1 (14.3%)
Other & Not Recorded	160 (16.5%)	85 (15.9%)	74 (17.3%)	1 (14.3%)
BMI (kg/m^2^)				
Average (median)	25.2 (24.2)	25.2 (24.2)	25.1 (24.3)	26.1 (20.2)
<18.5	97 (10.0%)	57 (10.7%)	37 (8.6%)	3 (42.9%)
18.5 - <20	64 (6.6%)	32 (6.0%)	32 (7.5%)	0 (0%)
20 - <25	292 (30.1%)	162 (30.3%)	128 (29.9%)	2 (28.6%)
25 - <30	222 (22.9%)	122 (22.8%)	100 (23.4%)	0 (0%)
≥30	138 (14.2%)	73 (13.6%)	63 (14.7%)	2 (28.6%)
Unknown	157 (16.2%)	89 (16.6%)	68 (15.9%)	0 (0%)

42.6% of the patients were male and 57.4% were female. Patients were divided into age groups of 18-49, 50-59, 60-69,70-79,80-89 and ≥90. The numbers of patients in each age group were 139 (14.3%), 102 (10.5%), 123 (12.7%), 243 (25.1%), 249 (25.7%), 114 (11.8%), respectively. The median age was 75 in the entire study population, 76 among those with fecal impaction, 74 among those with nonperforated stercoral colitis, and 69 among those with perforation. Racial demographics included 666 (69%) white, 144 (15%) black, and 160 (17%) patients of other/unknown race. Patients were divided into six groups by BMI, <18.5, 18.5-<20, 20-<25, 25-<30, ≥30, and unknown. The numbers of patients in each BMI group were 97 (10%), 64 (7%), 292 (30%), 222 (23%), 138 (14%) and 157 (16.2%), respectively. The average BMI was 25.2±6.7 kg/m^2^.

Outcomes

Outcomes are listed in Table 3.

**Table 3:** Outcomes Outcomes are given on an encounter basis, not as numbers of unique patients.  LOS, length of stay.  Table entries are formatted as N (%), except for the row "Encounters" and the LOS.  Percentages use the entries in "Encounters" as the denominators.

Outcome	Fecal Impaction + Nonperforated Stercoral Colitis, N (%)	Fecal Impaction, N (%)	Nonperforated Stercoral Colitis, N (%)	p-value (Perforated Cases Not Included)	Perforated, N (%)
Encounters	963	535	428		7
Admitted	797 (82.8%)	428 (80.0%)	369 (86.2%)	.011	7 (100%)
ICU stay	143 (14.8%)	78 (14.6%)	65 (15.2%)	.792	3 (42.9%)
Average LOS, days (admitted only)	8.8	9.0	8.6	.429	16.6
Died	30 (3.1%)	14 (2.6%)	16 (3.7%)	.320	3 (42.9%)
Discharged to hospice	97 (10.1%)	57 (10.7%)	40 (9.3%)	.503	2 (28.6%)
Combined died + discharged to hospice, all encounters	127 (13.2%)	71 (13.3%)	56 (13.1%)	.932	5 (71.4%)
Combined died + discharged to hospice, admitted only	124 (15.6%)	69 (16.1%)	55 (14.9%)	.637	5 (71.4%)

Among the 963 patients with fecal impaction or nonperforated stercoral colitis, 17.2% were discharged from the ED without hospital admission and 82.8% were admitted (fecal impaction: 80%; nonperforated stercoral colitis: 86.2%; p = 0.011). All seven patients with perforation were admitted. Among all ED encounters with fecal impaction or nonperforated stercoral colitis, 14.8% resulted in an ICU stay. Of those who were admitted, the average length of stay was 8.90 days (fecal impaction: 9.0 days; nonperforated stercoral colitis: 8.6 days; p = 0.43). The average length of stay among patients with perforation was 16.6 days.

Among the 963 patients with fecal impaction or nonperforated stercoral colitis, 30 (3.1%) died and 97 (10.1%) were discharged to hospice, for a combined risk of death or discharge to hospice 13.2%. There was no statistically significant difference in the length of stay, risk of death, discharge to hospice, or combined risk of death or discharge to hospice between the patients with fecal impaction and nonperforated stercoral colitis. Of the seven patients with perforation, seven were admitted, three went to the ICU, three died, and two were discharged to hospice. The relationships between death or discharge to hospice and age, body mass index, and time to diagnosis are shown in Table 4.

**Table 4:** Risk of death or discharge to hospice versus age, body mass index, and time to diagnosis Abbreviations: D/H, died or discharged to hospice; FI, fecal impaction; NSC, Nonperforated stercoral colitis; BMI, body mass index. Numerical entries are presented as: percent died or discharged to hospice (number in group). Statistics were performed on the aggregated group of fecal impaction and nonperforated stercoral colitis.

Risk of Death or Hospice	FI + NSC, % D/H (size of group)	Odds Ratio[95% CI], p-value	Fecal Impaction, % D/H (size of group)	Nonperforated Stercoral Colitis, % D/H (size of group)	Perforated, % D/H (size of group)
Age					
18-49	2.9% (N = 139)	Reference group	3.8% (N = 78)	1.6% (N = 61)	0% (N = 0)
50-59	5.9% (N = 102)	2.11[0.58,7.68], p = 0.257	3.8% (N = 52)	8.0% (N = 50)	0% (N = 0)
60-69	9.2% (N = 119)	3.43[1.07,11.01], p = 0.039	11.5% (N = 61)	6.9% (N = 58)	75.0% (N = 4)
70-79	15.7% (N = 242)	6.29[2.20,18.02], p < 0.001	13.1% (N = 122)	18.3% (N = 120)	100% (N = 1)
80-89	17.4% (N = 247)	7.11[2.50,20.08], p < 0.001	18.5% (N = 146)	15.8% (N = 101)	50.0% (N = 2)
≥90	21.9% (N = 114)	9.48[3.19,28.17], p < 0.001	21.1% (N = 76)	23.7% (N = 38)	0% (N = 0)
BMI (kg/m^2^)					
<18.5	23.4% (N = 94)	3.46[1.76,6.82], p < 0.001	26.3% (N = 57)	18.9% (N = 37)	133.3% (N = 3)
18.5 - <20	17.2% (N = 64)	2.35[1.05,5.288], p = 0.038	15.6% (N = 32)	18.8% (N = 32)	0% (N = 0)
20 - <25	13.8% (N = 290)	1.81[1.00,3.26], p = 0.047	14.8% (N = 162)	12.5% (N = 128)	100% (N = 2)
25 - <30	8.1% (N = 222)	Reference group	8.2% (N = 122)	8.0% (N = 100)	0% (N = 0)
≥30	11.0% (N = 136)	1.41[0.68,2.89], p = 0.355	12.3% (N = 73)	9.5% (N = 63)	100% (N = 2)
NR	13.4% (N = 157)	1.75[0.90,3.41]. p = 0.100	9.0% (N = 89)	19.1% (N = 68)	0% (N = 0)
Hours to diagnosis					
<3	8.0% (N = 336)	Reference group	7.1% (N = 183)	9.2% (N = 153)	50% (N = 2)
3 - <6	12.0% (N = 284)	1.53[0.90,2.61], p = 0.117	12.7% (N = 158)	11.1% (N = 126)	0% (N = 1)
6 - <12	16.2% (N = 74)	2.18[1.04,4.54], p = 0.037	18.9% (N = 37)	13.5% (N = 37)	0% (N = 0)
≥12	20.1% (N = 269)	2.83[1.73,4.63], p < 0.001	19.7% (N = 157)	20.5% (N = 112)	100% (N = 4)

The absolute risk of death or discharge to hospice rose approximately 4% per decade after age 50, increasing from 2.9% among patients with fecal impaction or nonperforated stercoral colitis aged 18-49 to 21.9% among patients aged 90 and older (p < 0.001). Compared to the reference group of age 18-49, all other age groups had significantly higher chances of death or discharge to hospice except the age group of 50-59. The odds ratios and 95% CIs with are age 60-69: 3.43[1.07,11.01], p = 0.039; age 70-79: 6.29[2.20,18.02], p < 0.001; age 80-89: 7.11[2.50,20.08], p < 0.001; and age ≥90: 9.48[3.19,28.17], p < 0.001.

Patients with fecal impaction or nonperforated stercoral colitis who had a BMI of 25-30 had a risk of death or discharge to hospice of 8.1% compared to 23.4% among those with a BMI of < 18.5 (p < 0.001), 17.2% among those with a BMI of 18.5-<20 (p = 0.038), and 13.8% among those with a BMI of 20-<25 (p = 0.047). The odds ratios with 95% CI are 3.46[1.76,6.82], 2.35[1.05,5.288], and 1.81[1.00,3.26] for BMI < 18.5, 18.5-<20 and 20-<25, respectively. Compared with patients with a body mass index of 25-<30, patients with a BMI of ≥30 and patients with unrecorded BMI did not have a significantly different risk of death or discharge to hospice.

The risk of death or discharge to hospice rose from 8.0% for patients diagnosed with fecal impaction or nonperforated stercoral colitis in less than three hours from triage to 20.1% among those diagnosed in ≥12 hours after triage (p < 0.001). Patients diagnosed in 3-<6 hours did not have a significantly different risk of death or discharge to hospice compared to patients diagnosed in <3 hours. Compared to the reference group of patients diagnosed in <3 hours, patients diagnosed in 6-<12 and ≥12 hours had significantly higher chances to death or discharge to hospice with odds ratios and 95% CIs of 2.18[1.04,4.54], p = 0.037, and 2.83[1.73,4.63], p < 0.001, respectively.

ICD-10 code analysis

Of the 72,836 valid ICD-10 codes recognized by CMS [17], 1,624 had at least one occurrence in our dataset. A median of 13 and an average of 13.7 valid ICD-10 codes were associated with each encounter. The most common code was K59.00 (constipation, unspecified), followed by K56.41 (fecal impaction). Only 36.6% of the encounters in our study were coded for fecal impaction (fecal impaction: 36.4%, nonperforated stercoral colitis: 36.7%, perforated 42.9%). There is no ICD-10 code that perfectly matches stercoral colitis; the closest approximations are K52.9 (noninfective gastroenteritis and colitis, unspecified), which occurred in 15% of all encounters (fecal impaction: 2.6%, stercoral colitis 30.1%, perforated 28.6%), and K52.89 (other specified noninfective gastroenteritis and colitis), which occurred in 6.4% of all encounters (fecal impaction: 0.4%, nonperforated stercoral colitis: 14.0%, perforated: 0%). The most appropriate code for stercoral ulcers (K62.6, ulcer of anus and rectum) was present in 2.7% (fecal impaction: 0.7%, nonperforated stercoral colitis: 5.1%, perforated: 0%). Select ICD-10 codes associated with an increased risk for death or discharge to hospice are shown in Table 5.

**Table 5:** Risk of death or discharge to hospice versus risk factors identified by ICD-10 codes Abbreviation: D/H, died or discharged to hospice.  ICD-10 codes were bundled into groups for sepsis, diabetes, dementia, decubitus ulcer, malnutrition, and paralysis/neuromuscular disease (“NMD”) as described in Methods and Table 1. Numerical entries are presented as: percent dead or discharged to hospice in a given group (number in group). Statistics were performed the aggregated group of fecal impaction and nonperforated stercoral colitis. Patients with at least one code for dementia, paralysis/NMD, or malnourishment were included in “Dementia or paralysis/NMD or malnutrition.”

	Fecal Impaction + Nonperforated Stercoral Colitis, % D/H (Number in Group)	p-value, Odds Ratio[95%CI]	Fecal Impaction, % D/H (Number in Group)	Nonperforated Stercoral Colitis, % D/H (Number in Group)	Perforated, % D/H (Number in Group)
Sepsis		p < 0.001			
Absent	8.1% (N = 704)	Reference group	9.0% (N = 391)	7.0% (N = 313)	100% (N = 2)
Present	27.0% (N = 259)	4.21[2.86,6.18]	25.0% (N = 144)	30.0% (N = 115)	60% (N = 5)
Diabetes		p = 0.038			
Absent	11.7% (N = 675)	Reference group	12.4% (N = 378)	10.8% (N = 297)	66.7% (N = 6)
Present	16.7% (N = 288)	1.51[1.02,2.23]	15.3% (N = 157)	18.3% (N = 131)	100% (N = 1)
Dementia		p < 0.001			
Absent	8.9% (N = 677)	Reference group	9.0% (N = 367)	8.7% (N = 310)	60% (N = 5)
Present	23.4% (N = 286)	3.15[2.15,4.61]	22.3% (N = 168)	24.6% (N = 118)	100% (N = 2)
Decubitus Ulcer					
Absent	11.2% (N = 885)	Reference group	11.5% (N = 496)	10.8% (N = 389)	60% (N = 5)
Present	35.9% (N = 78)	4.45[2.68,7.39]	35.9% (N = 39)	35.9% (N = 39)	100% (N = 2)
Malnutrition		p < 0.001			
Absent	8.7% (N = 711)	Reference group	9.7% (N = 393)	7.5% (N = 318)	50% (N = 4)
Present	25.8% (N = 252)	3.64[2.48,5.34]	23.2% (N = 142)	29.1% (N = 110)	100% (N = 2)
Paralysis/NMD		p < 0.001			
Absent	9.1% (N = 723)	Reference group	8.7% (N = 390)	9.6% (N = 333)	50% (N = 4)
Present	25.4% (N = 240)	3.34[2.31,4.99]	25.5% (N = 145)	25.3% (N = 95)	100% (N = 3)
Dementia or Paralysis/NMD or Malnutrition		p < 0.001			
Absent	1.9% (N = 413)	Reference group	2.3% (N = 215)	1.5% (N = 198)	33% (N = 3)
Present	21.6% (N = 550)	14.0[6.75,28.96]	20.6% (N = 320)	23.0% (N = 230)	100% (N = 4)

Among patients with fecal impaction or nonperforated stercoral colitis, 29.7% had codes for dementia and 23.4% of those patients died or were discharged to hospice; 24.9% had codes for paralysis or movement disorders and 25.4% died or were discharged to hospice; 8.1% had codes for decubitus ulcers and 35.9% died or were discharged to hospice; 26.2% had codes for malnutrition/failure to thrive and 25.8% died or were discharged to hospice; and 29.9% had codes for diabetes and 16.7% died or were discharged to hospice. The odds ratios with 95% CI are 4.21[2.86,6.18], 1.51[1.02,2.23], 3.15[2.15,4.61], 4.45[2.68,7.39], 3.64[2.48,5.34], 3.34[2.31,4.99] and 14.0[6.75,28.96] for patients with sepsis, diabetes, dementia, decubitus ulcers, malnutrition, paralysis/neuromuscular disease, and dementia or paralysis/neuromuscular disease or malnutrition, respectively.

Of the 550 patients with fecal impaction or nonperforated stercoral colitis and at least one of three key risk factors (dementia, paralysis/neuromuscular disease, or malnutrition/failure to thrive), 21.6% died or were discharged to hospice, compared to a risk death or discharge to hospice of 1.9% among patients with none of these risk factors (p < 0.001). Patients with at least one of these three risk factors accounted for 119 (93.7%) of the 127 deaths/discharges to hospice among patients with fecal impaction or nonperforated stercoral colitis.

An ICD-10 code for sepsis was present in 259 (26.9%) of patients with fecal impaction or nonperforated stercoral colitis, of whom 27.0% died or were discharged to hospice. Of the 30 patients with fecal impaction or nonperforated stercoral colitis who died, 27 (90.0%) had at least one code for sepsis. Of the 97 who were discharged to hospice, 43 (44.3%) had at least one code for sepsis.

Consults and procedures

The number of patients receiving various inpatient consultations and surgical procedures are presented in Table 6.

**Table 6:** Inpatient consults and procedures Abbreviations: GI, gastroenterology. GS/CRS, general surgery or colorectal surgery. ID, infectious diseases. EGD, esophagogastroduodenoscopy. EUA, rectal exam under anesthesia. Percentages are given as a percentage of patients admitted, not as a percentage of patients presenting to the ED.

	Fecal Impaction + Nonperforated Stercoral colitis, N (%)	Fecal Impaction, N (%)	Nonperforated stercoral colitis, N (%)	Perforated, N (%)
Number Admitted	797	428	369	7
Consult				
GI	372 (46.7%)	172 (40.2%)	200 (54.2%)	4 (57.1%)
GS/CRS	96 (24.6%)	84 (19.6%)	112 (30.4%)	7 (100%)
ID	326 (40.9%)	180 (42.1%)	146 (39.6%)	4 (57.1%)
Scopes				
EGD	89 (11.1%)	49 (11.4%)	40 (10.8%)	2 (28.6%)
Colonoscopy	77 (9.7%)	25 (5.8%)	52 (14.1%)	1 (14.3%)
Operations				
Any abdominal surgery	91 (11.4%)	41 (9.6%)	50 (13.6%)	4 (57.1%)
G-tube only	40 (5.0%)	22 (5.1%)	18 (4.9%)	0 (0%)
EUA + disimpaction only	13 (1.6%)	0 (0%)	13 (3.5%)	1 (14.3%)
Colon surgery	18 (2.3%)	4 (0.9%)	14 (3.8%)	3 (42.9%)

A gastroenterologist was consulted in 46.8% of the admissions. A general or colorectal surgeon was consulted in 25.2% of admissions. An infectious diseases specialist was consulted in 41.0%. Esophagogastroduodenoscopy was performed in 11.3% of those admitted. Colonoscopy was performed in 9.7% of those admitted. Any kind of abdominal surgery (as defined in Methods) was performed in 11.8% of those admitted. Gastrostomy tube placement was the only abdominal surgery in 5.0%. Rectal exam under anesthesia with disimpaction was performed in 1.7%.

Surgery on the colon other than rectal exam under anesthesia with disimpaction was performed in 2.6%. Among the four patients with fecal impaction who underwent surgery on the colon, three survived and one died. Among the 14 patients with nonperforated stercoral colitis who underwent surgery on the colon, 13 survived without discharge to hospice and one died. Among the three patients with perforation who underwent surgery on the colon, two survived and one died. Out the 35 patients who underwent either EUA or colon surgery, four (11.4%) died and none were discharged to hospice.

## Discussion

We found that fecal impaction and nonperforated stercoral colitis have equivalent outcomes and are both associated with a high risk of in-hospital mortality or discharge to hospice. We identified age ≥ 60, BMI < 25, dementia, paralysis or movement disorders, and malnutrition as key risk factors for poor outcomes. Diabetes is less strongly associated with death or discharge to hospice but is also a statistically significant risk factor. We showed that ICD-10 codes for sepsis are present in 90% of patients with fecal impaction and/or stercoral colitis who expire in the hospital. We also showed that the risk of in-hospital mortality or discharge to hospice increases if the diagnosis is not made in the first few hours after presentation. We confirmed that perforated stercoral colitis has a poor prognosis, with three deaths and two discharges to hospice among the seven patients with perforation in our study.

Ours is the first large cohort study to show that there is a high risk of mortality or discharge to hospice associated with fecal impaction even in the absence of stercoral colitis. The higher risk of mortality and discharge to hospice in our study compared to the Beirut study [11] is likely due to differences in patient populations and diagnostic modality (CT in our study, combinations of digital rectal exam, and unspecified imaging in the Beirut study). Like our study, however, the Beirut study also noted that short delays in care were associated with a higher risk of complications.

Our results with stercoral colitis are similar to the results published by Keim et al. [15]. Keim found that stercoral colitis was diagnosed on 0.31% of ED CT scans, compared to 0.21% of all inpatient and outpatient CT scans in our study. Keim found that 30-day all-cause mortality for patients admitted with nonperforated or perforated stercoral colitis was 10.8%, compared to a 16.0% combined risk of mortality or discharge to hospice in this study among patients admitted with either nonperforated or perforated stercoral colitis. The small difference between the studies probably reflects stochastic variation, the fact that not all patients on hospice die within 30 days, and differences in population and methodology. Keim included only CT scans ordered in the ED, whereas we included CTs obtained in the ED and CTs obtained after the patient had been admitted. The two studies provide large, independent, contemporaneous datasets drawn from tertiary and community hospitals in different geographical settings yielding similar results. This is a powerful confirmation, and it is likely that the numbers obtained in the two studies accurately represent the outcomes seen at a broad range of practices in the US.

We estimate that fecal impaction and stercoral colitis are associated with 124,500 ED visits annually, with about 16,000-17,000 of those encounters resulting in death or discharge to hospice. Our estimate derives from prior research showing 42,481 ED visits in 2011 with an ICD-9 code for fecal impaction [7], our finding is that the ICD-10 code for fecal impaction is present only in 36.6% of encounters with radiologically suspected fecal impaction/stercoral colitis, and a correction for interval US population growth (311.6 million to 334.2 million) from 2011 to 2023. Assuming that patients discharged to hospice die a relatively short time later, approximately 0.4%-0.5% of the 3.5 million annual deaths in the US [18] are associated with (although not necessarily caused by) fecal impaction or stercoral colitis. Many deaths associated with fecal impaction or nonperforated stercoral colitis are likely to be preventable.

We were surprised at the number of upper endoscopies in our study. Some of the upper endoscopies and gastrostomies may have been prompted by nausea, vomiting, hematemesis, poor oral intake, and/or adult failure to thrive secondary to fecal impaction instead of upper GI tract pathology. We predict that prompt and effective treatment of fecal impaction/stercoral colitis would reduce the number of upper endoscopies and also reduce the number of gastrostomy tube placements.

The extent to which fecal impaction and nonperforated stercoral colitis are causal factors in the poor outcomes we observed is unclear. We believe that patients with CT scans meeting the study criteria fall into three groups: (1) a small minority in whom fecal impaction and/or stercoral colitis are the obvious primary cause of a patient’s death or discharge to hospice; (2) a larger minority in whom another serious problem such as stroke or advanced cancer has left the patient in a moribund condition and in whom the fecal impaction/stercoral colitis has no material impact on the patient’s terminal course; and (3) a large plurality or even a majority of cases in which a patient’s fecal impaction/stercoral colitis is a significant contributory factor, along with other serious comorbidities, to a cascade of events that result in death or discharge to hospice. Both Sommers and Keim estimated that approximately 50% of the deaths they observed were secondary to fecal impaction or stercoral colitis.

Concordant with prior autopsy data [3,19], we suspect that stercoral ulcers and colonic epithelial injuries too small or shallow to visualize on CT are ubiquitous among patients with fecal impaction/stercoral colitis. We think that mucosal damage associated with fecal impaction enables bacterial translocation and accounts for the high frequency with which sepsis was observed in our patient population. We believe that some of the 331 patients diagnosed with urinary tract infections and 105 patients diagnosed with pneumonia in our study were actually septic due to fecal impaction/stercoral colitis and that the source of sepsis was incorrectly attributed to the urinary or respiratory tract. We hypothesize that sepsis due to bacterial translocation via small stercoral ulcers and colonic epithelial injuries is the ultimate cause of death in many patients with fecal impaction and/or stercoral colitis even in the absence of perforation.

Limitations

Our study was a retrospective chart review, so we cannot know if fecal impaction and stercoral colitis cause the poor outcomes we observed or are simply markers of overall mortality risk. Our quantitative body mass index data should be interpreted cautiously since many of these values were estimated, not measured, by the triage provider and had poor precision compared to values obtained at later in the same hospitalization or on subsequent encounters. We included CT reports that indicated any nonzero probability of fecal impaction or stercoral colitis; many interpreting radiologists used nebulous language that made it difficult to ascertain their confidence. We almost definitely undercounted the number of perforations secondary to fecal impaction and stercoral colitis because any report identifying a colonic perforation without explicitly naming fecal impaction or stercoral colitis as the underlying cause would not have been retrieved in our keyword search. Our time-to-diagnosis data did not differentiate delayed diagnoses that were present at the time of presentation from cases of fecal impaction and stercoral colitis that developed during hospitalization. Our study also has a selection bias: we only know the outcomes of patients who presented to the ED, received CT scans and had not been diagnosed by a different modality. Outpatients and ED patients/inpatients who were diagnosed based on clinical exams or other imaging modalities (primarily radiographs) may have outcomes different than those we report.

## Conclusions

Fecal impaction and nonperforated stercoral colitis are associated with a high risk of death or discharge to hospice, particularly among patients over age 60 or with low BMI, dementia, paralysis/neuromuscular disease, or malnutrition. We suspect that fecal impaction and nonperforated stercoral colitis contribute to and are not merely correlated with many of the poor outcomes we observed. Diagnosis within the first few hours after presentation may reduce mortality.
